# The impact of COVID-19 pandemic on nosocomial infections in the cardiac care unit of a non-epidemic hospital in China

**DOI:** 10.3389/fmed.2025.1483967

**Published:** 2025-01-20

**Authors:** Xiang Liao, Wei Wu, Lijuan Zhang, Zheng Zhang, Chengrong Zheng, Xincheng Qiu, Chao Xin, Zhitao Jin

**Affiliations:** ^1^Department of Cardiology, PLA Rocket Force Characteristic Medical Center, Beijing, China; ^2^Department of Anaesthesia and Surgery Centre, Sichuan Provincial People's Hospital, School of Medicine, University of Electronic Science and Technology of China, Chengdu, China

**Keywords:** nosocomial infections, cardiac care units, COVID-19 pandemic, non-epidemic hospital, China

## Abstract

**Background:**

COVID-19 is generally believed to increase the risk of nosocomial infections, however, there is a gap in relevant researches on critically ill patients in cardiac care units (CCU).

**Method:**

This cross-sectional research was conducted in a tertiary-level non-epidemic hospital of Beijing, capital of China. The nosocomial infection rates of CCU were assessed prior to and during the of COVID-19 outbreak.

**Results:**

During the COVID-19 pandemic, the overall incidence of nosocomial infections decreased by 20.6-percent compared with the pre - pandemic period. Specifically, the total nosocomial infection rate during the COVID-19 pandemic (*p* = 0.04) decreased by 20.6%. Among various types of CCU-acquired nosocomial infections, the rates of pneumonia, urinary tract infection (UTI), bloodstream infection (BSI), gastrointestinal infection, and skin infection decreased by ranges from 4.7 to 100% during the COVID-19 pandemic. Meanwhile, a 1.5-percent increase in ventilator-associated events (VAEs) was observed during the COVID-19 pandemic.

**Conclusion:**

During the COVID-19 pandemic, stricter implementation of infection control protocols appears to reduce nosocomial infections in CCU.

## Introduction

The COVID-19 outbreak in rapidly spread from Wuhan at the end of 2019. A study involving 72,314 cases revealed that there were 889 asymptomatic patients, accounting for approximately 1% (1). COVID-19 is characterized by human-to-human transmission in settings such as hospitals and households, and it has been categorized as a Category B infectious disease yet managed in accordance with Category A infectious disease protocols (2). Although the COVID-19 pandemic has heightened the awareness of infection control among the public and medical staff (3), it has also negatively impacted certain infection control practices (4). For instance, patients seeking medical treatment in hospitals present with more severe conditions, necessitating more invasive therapies like ventilators and intravenous catheters (5). Despite the strict implementation of infection control measures in most healthcare facilities, COVID-19 patients remain at a high risk of secondary bacterial infections and nosocomial infections due to long-term illness, extended hospitalization, the use of immunosuppressive drugs, and increased utilization of medical devices (6, 7).

During the pandemic, emergency trauma patients still necessitated hospitalization in intensive care units, and the possibility of asymptomatic infection could not be precluded. Owing to the critical condition of these patients, their attenuated physical functions, compromised immunity, and diverse iatrogenic factors, patients in cardiovascular intensive care units have invariably been a high-risk population for nosocomial infections (4). Nosocomial infections not only augment the affliction and economic burden of patients but also exert a severe impact their recovery and life safety. To forestall hospital-acquired infections, numerous medical centers have adopted standard infection control modalities, encompassing rational allocation of equipment, triage strategies to alleviate congestion, reorganization of wards and environmental hygiene, as well as training and enforcement of standard precautions, such as safe injection practices, hand and personal hygiene (8, 9).

In comparison with high-risk areas for cross-infection such as Hubei Province, the overall risk of cross-infection in Beijing was relatively low (10). The primary reason why Beijing was not significantly affected by the epidemic can be largely attributed to the stringent preventive measures implemented by the local government during the pandemic. These measures included wearing masks, the closure of public venues such as restaurants, the promotion of remote work, the prohibition of gatherings, as well as regular nucleic acid testing (11). In light of the potential risk of cross-infection, our hospital, similar to the majority of hospitals in Beijing, suspended percutaneous coronary intervention (PCI), which consequently led to a significant decrease in the number of CCU patients during the same period (12).

Nevertheless, there exists a contradiction regarding what is commonly known about the impact of COVID-19 prevention and control measures on nosocomial infections. Some studies reported a reduction in nosocomial infection within specific wards, such as the neurologic ward, while have presented contrary findings (13, 14). The objective e of the study was to investigate the impact of the COVID-l9 epidemic on the rate of nosocomial infections in the adult cardiac care units (CCU) of a tertiary non-epidemic hospital in Beijing, China.

## Materials and methods

### Study design and sampling

A cross-sectional study design was employed to examine the nosocomial infection rate both during and before the COVID-19 pandemic within the CCU of a tertiary hospital in Beijing. The PLA Rocket Force Characteristic Medical Center, which is a tertiary hospital in proximity to the center of Beijing, was selected as the research site. The CCU of the Department of Cardiology in this hospital is equipped with 10 beds. Meanwhile, it is furnished with advanced treatment equipment and staffed by a highly skilled cardiac intensive care team. This study retrospectively collected data regarding the occurrence of nosocomial infections among all CCU patients during the initial four months of the COVID-19 pandemic in 2020 (January 24, 2020 to May 24, 2020), as well as during the corresponding period before the COVID-19 pandemic (January 24, 2019 to May 24, 2019).

Only patients presenting symptoms related to nosocomial infection and with pathogens detected through diverse biological samples were deemed eligible for inclusion in this study. All relevant data were recorded in the nosocomial infection surveillance system by a dedicated infection control team. Typically, this team consists of an infection control physician and a nurse, and is tasked with excluding data that are either unqualified or incomplete. Infection control team determines the group categorization of patients with nosocomial infections, including pneumonia (PNEU), urinary tract infection (UTI), ventilator-associated events (VAEs), gastrointestinal infection, bloodstream infection (BSI) and skin infection. Upon obtaining the approval of the hospital ethics committee, the researchers collected the data with nosocomial infection rate in CCU from the hospital infection control department.

### Statistical analysis

According to the normal distribution, continuous variables were presented in the form of mean ± standard deviation. Meanwhile, a Student’s t-test was conducted as appropriate at the same time. The incidence of the nosocomial infection rate within CCU during and prior to the COVID-19 pandemic was expressed in terms of frequency and percentage. The percentage change was calculated by (((new value in 2020 - old value in 2019)/old value in 2019) ∗ 100), which was employed to describe the variations in the nosocomial infection rate associated with COVID-19 in the cardiac care unit in China. Furthermore, count data were described as cases (%). Pearson’s chi-square test (χ^2^ test) was utilized to compare the changes in infection rate between different groups. As is standard practice, SPSS 20 was applied in all statistical analyses, and a *p* < 0.05 was regarded as statistically significant.

## Results

### General patient information

A total of 28 cases of CCU patient admissions were documented during the initial four months of the COVID-19 outbreak (January 24, 2020 to May 24, 2020), and 48 cases at the same period in the year 2019 (January 24, 2019 to May 24, 2019). No statistical differences in general patient information, including age, sex, body mass index (BMI), hypertension, diabetes mellitus, COPD, cigarettes, and advanced age (≥80), and inflammatory biomarkers [procalcitonin (PCT),C-reactive protein (CRP) and interleukin 6 (IL-6)] of the 1st day between the two groups (Table 1).

**Table 1:** Clinical characteristics of the patient.

		The sampling period of the year during COVID-19 (*n* = 28)	The sampling period of the year before COVID-19 (*n* = 48)	*t*/*χ*^2^	p
Age		82.93 ± 7.72	80.54 ± 13.00	0.883	0.38
Sex	Male	22	30	0.926	0.336
	Female	7	18		
BMI		23.52 ± 2.77	23.35 ± 3.1	0.246	0.806
Hypertension, n (%)		22 (78.6)	40 (83.3)		>0.05
Diabetes mellitus, *n* (%)		8 (28.6)	17 (35.4)		>0.05
COPD, n (%)		3 (10.7)	4 (8.3)		>0.05
Cigarettes, n (%)		10 (35.7)	14 (27.1)		>0.05
Advanced age, n (%)		21 (75)	33 (68.8)		>0.05
PCT (ng/mL)		0.236 ± 0.125	0.214 ± 0.113		>0.05
CRP (mg/L)		16.4 ± 4.8	20.1 ± 5.9		>0.05
IL-6 (pg/L)		30.1 ± 7.1	32.9 ± 6.7		>0.05

### Comparison of nosocomial infection rates before and during COVID-19 pandemic

During the initial four months of the COVID-19 outbreak (January 24, 2020 to May 24, 2020), the overall rate of nosocomial infection was determined to be 10.7%. In contrast, during the corresponding period in the year 2019 (January 24, 2019 to May 24, 2019), this rate was 31.3%. Moreover, it was found that the nosocomial infection rate decreased by 20.6 percent during the pandemic in comparison to the pre- pandemic period (*p* = 0.04). With the exception of for VAEs, the rates of pneumonia, UTI,BSI, gastrointestinal infection, skin infection in 2020 all exhibited percentage changes when compared with the same term in 2019. However, no statistically vital differences were observed (*p* > 0.05). Intriguingly, there were no occurrences of bloodstream infection, gastrointestinal infection or skin infections during this period of pandemic. Furthermore, the rate of VAEs during the COVID-19 pandemic increased by 15 percent relative to the previous year. Once again, no statistical difference was observed (Table 2).

**Table 2:** Comparison of nosocomial infection rates before and during COVID-19 pandemic.

Variable	The sampling period of the year during COVID-19 (*n* = 28) *N* (%)	The sampling period of the year before COVID-19 (*n* = 48) *N* (%)	Percentage changes	*χ*^2^ (P)
Ventilator-associated events (VAEs)	1 (3.6)	1 (2.1)	1.5	0.15 (0.7)
Pneumonia (PNEU)	1 (3.6)	6 (3.6)	−8.9	0.79 (0.38)
Urinary tract infection (UTI)	1 (3.6)	4 (8.3)	−4.7	0.1 1 (0.74)
Bloodstream infection (BSI)	0	1 (2.1)	—	
Gastrointestinal infection	0	1 (2.1)	—	
Skin infection	0	2 (4.2)	—	
Total	3 (10.7)	15 (31.3)	−20.6	4.13 (0.04)

When comparing the laboratories, and inflammatory markers at CCU admission, we found that several biomarkers of systemic inflammation differed on 7th day among the study groups (Table 3). Significantly elevated levels of PCT were detected in subjects with nosocomial infected patient outcomes during hospitalization. The comparative analysis of the groups, no significant difference in the four indicators included IL-6, CRP, D- dimer, and WBC.

**Table 3:** Laboratories, and inflammatory markers at CCU admission, stratified by the nosocomial infection groups on the 7th day after CCU admission.

Biomaker on the 7th day after admission	The sampling period of the year during COVID-19 (*n* = 3)	The sampling period of the year before COVID-19 (*n* = 15)	*p*
IL-6 (pg/L)	126.4 ± 13.5	144.8 ± 19.7	0.146
CRP (mg/L)	38.8 ± 6.57	54.3 ± 4.52	0.11
PCT (ng/mL)	1.21 ± 0.37	2.09 ± 0.53	<0.05
D-dimer (ng/mL)	88.4 ± 7.29	102.1 ± 11.57	0.067
White blood cells (WBC, ×10^9^/L)	12.1 ± 2.57	12.8 ± 3.5	0.749

## Discussion

The present study demonstrated that there was a decline in the number of admissions to the CCU, with a decrease of approximately 40%. The strict implementation of public health measures has played a crucial role in controlling the COVID-19 outbreak, and this has been particularly evident in China However, it did exert a detrimental effect on non-epidemic hospital admissions. Furthermore, precise instructions regarding home confinement and the fear of being infected in medical centers could also deter individuals from seeking medical treatment in hospitals (15). From January 24, 2020 to May 24, 2020, Beijing witnessed the first peak of the epidemic and has implemented strict public health measures, such as home isolation. Meanwhile, our medical center also rigorously enforced norms that surpassed routine standards to prevent nosocomial infections. Consequently, we compared the variations during the initial four months of epidemic in Beijing with those in the corresponding period in 2019.

Nevertheless, a study conducted in Beijing indicated that the number of patients hospitalized in CCU witnessed an increase by 8.9%, but these differences did reach statistical significance (10). In alignment with previous data, pneumonia, urinary tract infections, and primary bloodstream infections were the most prevalent in the findings of the present study, both before and during the epidemic (16). Despite the existence of the possibility of asymptomatic infections and the presence of false-negative patients who were not screened, the number of patients with VAEs and pneumonia did show an upward trend, but no significant difference was observed. PCT is a classical early biochemical marker of bacterial infection, and an elevated serum level was positively correlated with the severity of infection (17). PCT has higher sensitivity and specificity for Gram-negative bacteria (18, 19). Significantly higher PCT level on 7th day was observed in individuals with nosocomial infections during 2020. This is consistent with the decrease in the number of patients with UTI and pneumonia, which are mainly caused by gram-negative bacteria. These findings suggest that PCT may be a common and useful biomarkers for predicting nosocomial infections in CCU patients (20–22).

Nosocomial infections are mostly prevalent in the CCU and in older adults with underlying diseases and suppressed immune system (8, 9). Therefore, they cause several problems in the treatment course of patients, including the high cost of laboratory procedures, the use of medication, the length of hospital stay, and antibiotic resistance (23). Applying contact precautions can reduce the incidence of nosocomial infections (24). Attributed to the development and widespread use of new broad-spectrum antibiotics, the prevalence and mortality rates of hospital-acquired infections, especially multi-drug resistant bacterial infections, show an increasing trend year by year. Hand hygiene has now become one of the most effective methods in fighting hospital-acquired infections. The hands of hospital staff are a key transmission medium for hospital infections. Through handwashing is the most direct, simple, economical, and effective measure to prevent hospital-acquired infections (25). Moreover, the raise of the awareness is undoubtedly one of the most effective methods of avoiding nosocomial infections, during the COVID-19 pandemic, we have conducted more stringent hand hygiene education and assessments for medical personnel and caregivers. The compliance with hand hygiene and the pass rate of surface bacterial counts on hands have improved significantly, effectively reducing the occurrence of hospital-acquired infections (15, 26).

During the early months of COVID-19, when health protocols were observed better, and the risk of nosocomial infections could be reduced by restricting family visits (27). The CCU adopted a restrictive visiting policy, advocating for non-family visits. If visits were necessary, they were limited to once per day, 15–30 min per visit, and only one visitor was allowed. Visitors were required to wear isolation gowns, N95 masks, practice hand hygiene, and maintain a distance, prohibiting close contact and touching various instruments. Additionally, a dedicated caregiver system was established in the CCU to reduce interactions with caregivers from general wards (28).Increased length of visit and frequency of visits increases the nosocomial infection rate, while increased frequency of sterilizing the facilities and the air decreases the nosocomial infection rate. Therefore, restricted visiting system is better than flexible visit system (29).

The present study has some limitations. The study relies on a small sample size with single center, which undermines the reliability and generalizability of the findings. In future studies, more representative sample is essential for drawing meaningful conclusions. The study on nosocomial infection rates of CCU has been conducted in a non-epidemic hospital of Beijing, China, which did not admit patients with COVID-19 diagnosis. We do not know if the rate of nosocomial infections will be different in patients with COVID-19. Furthermore, we also cannot rule out latent or asymptomatic infections of COVID-19. Therefore, more attention should be paid on the generalizability of these results, and more serious consideration should be given in the future studies.

## Data Availability

The datasets presented in this study can be found in online repositories. The names of the repository/repositories and accession number(s) can be found in the article/Supplementary material.
